# Impact of photobiomodulation therapy on pro-inflammation functionality of human peripheral blood mononuclear cells – a preliminary study

**DOI:** 10.1038/s41598-024-74533-y

**Published:** 2024-10-04

**Authors:** Kamila Pasternak-Mnich, Jolanta Kujawa, Justyna Agier, Elżbieta Kozłowska

**Affiliations:** 1 Department of Medical Rehabilitation, Faculty of Health Sciences, Medical University of Lodz, 251 Pomorska St, Lodz, 92-213 Poland; 2 Department of Microbiology, Genetics and Experimental Immunology, Lodz Centre of Molecular Studies on Civilisation Diseases, Medical University of Lodz, Lodz, 92-215 Poland

**Keywords:** Photobiomodulation Therapy (PBMT), Low-level laser therapy (LLLT), MLS M1 system, Peripheral blood mononuclear cells (PBMCs), Inflammation, Cytokines, Cell biology, Immunology

## Abstract

Research into the efficacy of photobiomodulation therapy (PBMT) in reducing inflammation has been ongoing for years, but standards for irradiation methodology still need to be developed. This study aimed to test whether PBMT stimulates in vitro human peripheral blood mononuclear cells (PBMCs) to synthesize pro-inflammatory cytokines, including chemokines. PBMCs were irradiated with laser radiation at two wavelengths simultaneously (λ = 808 nm in continuous emission and λ = 905 nm in pulsed emission). The laser radiation energy was dosed in one dose as a whole (5 J, 15 J, 20 J) or in a fractionated way (5 J + 15 J and 15 J + 5 J) with a frequency of 500, 1,500 and 2,000 Hz. The surface power densities were 177, 214 and 230 mW/cm^2^, respectively. A pro-inflammatory effect was observed at both the transcript and protein levels for IL-1β after PBMT at the energy doses 5 J and 20 J (ƒ=500 Hz) and only at the transcript level after application of PBMT at energy doses of 20 J (ƒ= 1,500; ƒ=2,000 Hz) and 5 + 15 J (ƒ=500 Hz). An increase in CCL2 and CCL3 mRNA expression was observed after PBMT at 5 + 15 J (ƒ=1,500 Hz) and 15 + 5 J (ƒ=2,000 Hz) and CCL3 concentration after application of an energy dose of 15 J (frequency of 500 Hz). Even though PBMT can induce mRNA synthesis and stimulate PBMCs to produce selected pro-inflammatory cytokines and chemokines, it is necessary to elucidate the impact of the simultaneous emission of two wavelengths on the inflammatory response mechanisms.

## Background

Peripheral blood mononuclear cells (PBMCs) are the main source of cytokines/chemokines found in the blood. According to the researchers, the reactivity of these cells can be altered in certain diseases, such as osteoarthritis, systemic lupus erythematosus or Parkinson’s disease. Cytokines influence the proliferation and differentiation and regulate the activity of many cells in the body^1^. Furthermore, they direct the course of both physiological and pathological processes and play a pivotal role in inflammatory processes. Studies over the past decade have demonstrated that the administration of laser radiation has enhanced the proliferation of PBMCs, particularly CD45^+^ lymphocytes and natural killer (NK) cells. Additionally, it has been observed to influence prostaglandin E2 (PGE2) levels in PBMCs subjected to inflammatory conditions^2,3^. The immunological response to damaging factors, namely inflammation, is the primary mechanism by which the body eliminates these agents and repairs injured tissues. Tissue damage stimulates the production of cytokines and chemokines by cells associated with the inflammatory response. Many factors can initiate and interrupt this complex process at various stages^4^. From a therapeutic perspective, it is particularly beneficial to employ pharmacological and non-pharmacological strategies to reduce the production of inflammatory mediators. Photobiomodulation therapy (PBMT) represents an alternative to pharmacological agents, and research into its effectiveness in reducing inflammation has been ongoing for years^5–10^. However, despite this, it has not yet been possible to develop standards for the methodology of applying laser radiation. Indeed, many authors have asserted that, from a therapeutic point of view, the anti-inflammatory effect of laser radiation is one of its most important biological effects^11–14^. Pro-inflammatory cytokines/chemokines that play a role in the pathogenesis of rheumatoid arthritis (RA), for which PBMT is used in the clinical setting as a treatment option, were selected for the study. The mechanism of action of PBMT has been identified as twofold. Firstly, the response to PBMT at the cellular level occurs due to the capture of photons by mitochondrial cytochromes c. This increases the production of cytochrome C oxidase and the synthesis of adenosine-5′-triphosphate (ATP), nitric oxide, reactive oxygen species (ROS) or calcium ions (Ca^2+^)^15–21^. PBMT indirectly or directly influences epigenetic mechanisms, including DNA synthesis, gene expression, and relative mRNA levels^22,23^. It has been demonstrated that PBMT reverses cognitive deficits in Alzheimer’s disease (AD) by promoting neurogenesis through the stimulation of the expression of interferon γ/interleukin 10 (IFN-γ/IL-10) in T lymphocytes^24^. Furthermore, studies have demonstrated that PBMT reduces oxidative stress and supports the proliferation of CD8^+^ T cells, thereby enhancing anti-tumor immunity^25^. It was also shown that laser stimulation of PBMCs resulted in an increase of IL-10 and the reduction of IFN-γ by these cells, which reduced nitrosative stress in multiple sclerosis (MS) patients^26,27^. It also proved that PBMT reduced inflammation and improved lung function in chronic airway inflammation by decreasing cytokine production and increasing the number of T lymphocytes^28^. A review of the literature revealed that laser radiation with a wavelength 405, 532, 635, 650, 670, 735, 810 and 830 nm reduced the expression of tumor necrosis factor (TNF-α), IL-1β, and IL-8^29,30^. The researchers demonstrated that administering 810 nm laser radiation with a power density of 16.7 mW/cm², delivered continuously, resulted in a fluence-dependent reduction (5 J/cm²) in the mRNA expression of pro-inflammatory cytokines^31^. Hwang demonstrated that the application of laser radiation at a specific wavelength (405 nm) and for a defined exposure time (48, 72 and 96 h) resulted in a change in IL-6 levels and exposure time-dependent alterations in IL-8 levels (exposure time of 48, 72 and 96 h). Pilar’s team, in a comprehensive systematic review, analyzed nearly 50 studies and showed that PBMT increased the synthesis of IL-33, TNF, IL-1β and acted as an inhibitor for chemokine (C-C motif) ligand (CCL): 5, 7, 17, 20; IL: 5, 6, 7, 9 and 10^32^. The researchers indicated that the wavelengths that affected cytokine production were primarily 655 and 660 nm and 606 + 808 nm. In contrast, radiation in the 665 and 810 nm range affected gene expression for selected cytokines. Also, Thome Lim’s team showed that the application of simultaneous continuous emission of two wavelengths of radiation (660 and 808 nm) with a fluence of 142.8 J/cm^2^ increased the relative expression of IL-1β mRNA and decreased the expression of mRNA for IL-10^33^. However, authors rarely indicate a detailed relationship between cytokine levels or mRNA expression of pro-inflammatory cytokines and the laser radiation parameters used. The most common indication is the dependence on the wavelength of radiation. Even though numerous researchers have discussed the mechanisms of action of low-energy laser radiation, the results remain controversial. The extensive range of possibilities for applying different laser emission sources, their parameters (wavelength, frequency, surface energy density and power), dosing methods (continuous or fractionated) and the simultaneous application of several wavelengths of radiation pose difficulties in the standardization of irradiation. The MLS M1 system was used during the study, which simultaneously emitted laser radiation of two distinct wavelengths (λ = 808 nm and λ = 905 nm). With this technological solution, it is possible to achieve different penetration depths of radiation and minimize the thermal effect through OFF and ON emission periods. As demonstrated in previous studies conducted by our research group, the MLS M1 system can influence several cellular processes, including morphology, viability, proliferation, intracellular Ca^2+^ concentration, free radical generation, apoptosis/necrosis, membrane fluidity, secondary structure of proteins and activity of membrane ATPases. Nevertheless, there is a lack of knowledge regarding the impact of MLS M1 system irradiation on the production of pro-inflammatory cytokines/chemokines. Research on the impact of PBMT on PBMCs can provide valuable information on molecular and cellular mechanisms, which cause this therapy to optimize its parameters, such as wavelength, light intensity, or exposure time, adapted to the patient’s specific needs. This is especially important for patients with weakened or impaired immune system function due to age (geriatrics) or chronic diseases (e.g., RA). Our study aimed to investigate whether PBMT, using laser radiation at two simultaneous wavelengths (808 nm in continuous emission and 905 nm in pulsed emission), can stimulate human PBMCs in vitro to synthesize pro-inflammatory cytokines, including chemokines (CCL2, CCL3, IL-1β, and IL-33). The selection of cytokines and chemokines for this study was based on their pivotal roles in inflammatory and immune responses. IL-1β is a critical pro-inflammatory cytokine in initiating and regulating the inflammatory response. While it can drive inflammation’s progression, this can be beneficial in acute phases but potentially harmful if dysregulated^34^. CCL2 is crucial for recruiting immune cells to sites of inflammation. It plays a significant role in tissue repair and the development of chronic inflammatory conditions^35^. CCL3, another important chemokine, recruits various immune cells, including macrophages and T cells, to inflammation sites and is often associated with inflammatory responses and tissue remodeling^36^. Similarly, IL-33 acts as an alarmin, signaling tissue damage and promoting inflammatory responses^37^. Each of these molecules is linked to various aspects of inflammation and tissue repair, making them relevant targets for evaluating the effects of PBMT. The study sought to determine the impact of different energy doses (5 J, 15 J, 20 J, and fractionated doses 5 J + 15 J and 15 J + 5 J) and frequencies (500; 1,500, and 2,000 Hz) on the production of these inflammatory markers, both at the transcript and protein levels, to better understand the mechanisms through which PBMT may influence the inflammatory response.

## Results

### The effect of PBMT on PBMC cytokine/chemokine mRNA expression

**Fig. 1:**
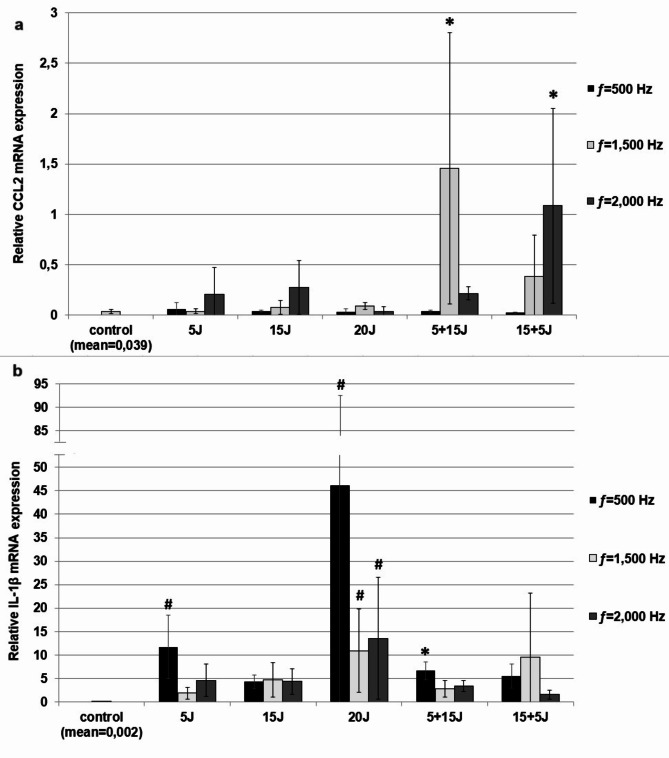
The effect of PBMT on (a) CCL3 and (b) IL-33 mRNA expression in PBMCs was evaluated using qRT-PCR. Irradiated PBMCs were cultured for 72 h at 1 × 10^6^ cells per well. Non-irradiated (no-treated) cells were used as control and cultured under the same conditions. Subsequently, PBMCs from 5 wells were pooled, and RNA was extracted from 5 × 106 PBMCs, converted into cDNA, and analyzed by qRT-PCR. The expression levels of genes encoding selected cytokines/chemokines were normalized to the housekeeping gene human ACTIN transcript level. Results are presented as the mean ± SD of three independent replicates. Statistical significance is indicated as *p < 0.05 and #*p* < 0.01.

**Fig. 2:**
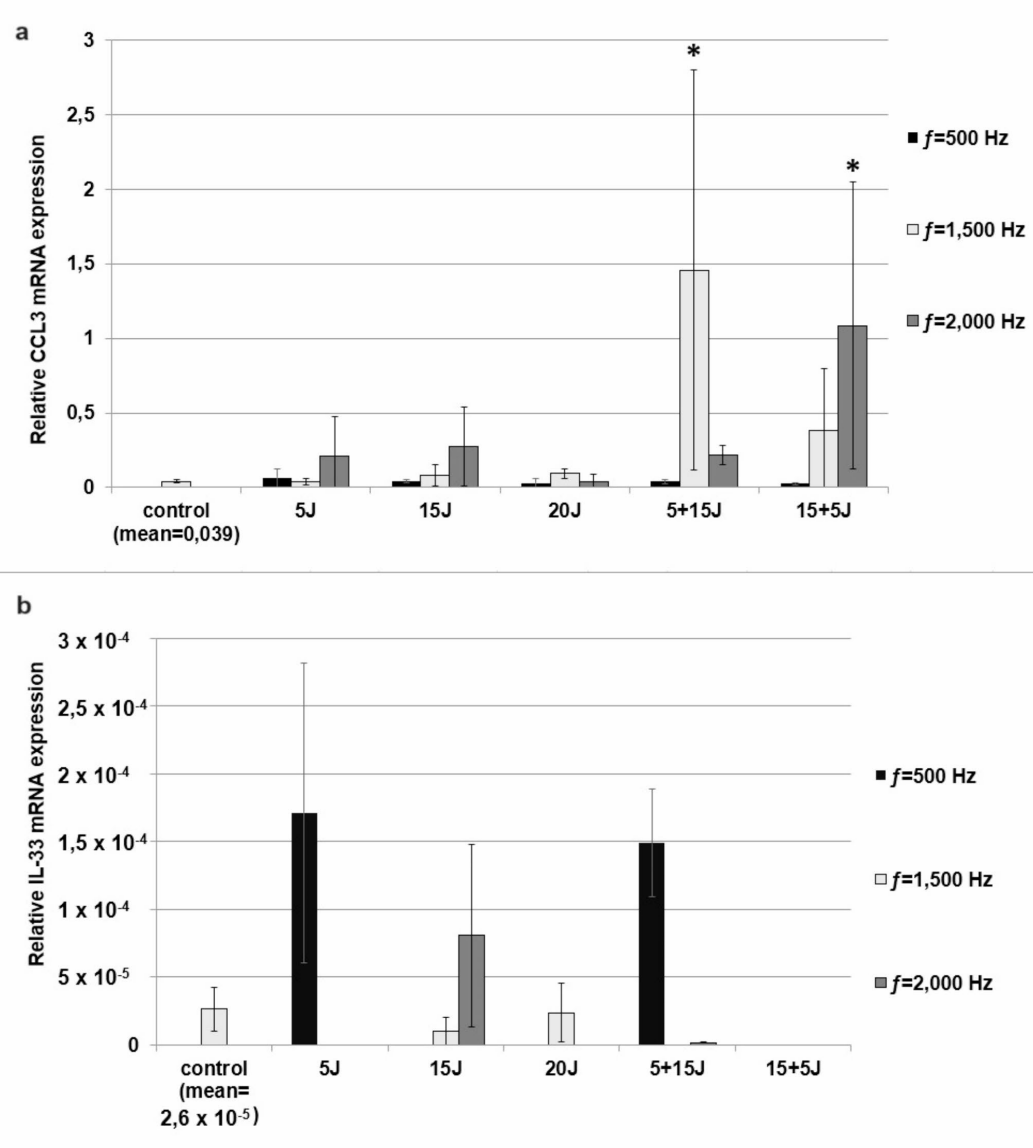
Effect of PBMT on (a) CCL-3, (b) IL-33 mRNA expression in PBMCs evaluated by qRT-PCR. PBMCs after irradiation were cultured for 72 h at concentrations of 1 × 10^6^ cells/mL (control was not irradiated). Afterwards, total mRNA was extracted and converted into cDNA, followed by qRT-PCR assay. The expression of genes encoding selected cytokines/chemokines was corrected by normalization based on the transcript level of the housekeeping gene human ACTIN. Results are shown as the mean ± SD of three separate experiments. *p < 0.05, ^**#**^*p* < 0.01.

The cytokine/chemokine mRNA expression in PBMCs was evaluated by qRT-PCR and presented as a relative cytokine/chemokine mRNA expression in laser irradiation-stimulated PBMCs compared to unstimulated cells. As presented in Figs. 1a and b and 2a and a statistically significant increase in CCL2, IL-1β and CCL3 mRNA expression in stimulated PBMCs was documented. The CCL2 mRNA expression level was statistically higher in cells stimulated by laser irradiation with energy dose 5 + 15 J (ƒ=1,500 Hz, mean ± SD: 1,457 ± 1,343, *p* = 0,0363) and 15 + 5 J (ƒ=2,000 Hz, mean ± SD: 1,086 ± 0,965, *p* = 0,0394) compared to the control. There was also an increase in IL-1β mRNA expression in PBMCs after laser irradiation at doses: 5 J (11,753 ± 6,729, *p* = 0,001), 20 J (46,073 ± 46,487, *p* = 0,000009) and 5 + 15 J (6,653 ± 1,876, *p* = 0,018) for ƒ=500 Hz and 20 J for ƒ=1,500 and 2,000 Hz (respectively: 10,939 ± 8,909, *p* = 0,004; 13,594 ± 12,94, *p* = 0,0008). Furthermore, the PBMT has been demonstrated to stimulate CCL3 mRNA expression. This effect was observed following exposure of the cells to irradiation under the following parameters: 5 + 15 J for ƒ=1,500 Hz (1,457 ± 1,343, *p* = 0,041) and 15 + 5 J for ƒ=2,000 Hz (1,086 ± 0,965; *p* = 0,045) (Fig. 2a). For IL-33 expression in PBMCs, numerical values were obtained only for some laser radiation parameters and were presented in Fig. 2b; for the rest, a result of “NEG (Multi Ct)” was obtained. Consequently, the effect of PBMT on IL-33 synthesis in PBMCs was not evaluated.

### The effect of PBMT on PBMC cytokine/chemokine synthesis

We evaluated whether PBMT stimulates PBMCs to produce selected pro-inflammatory cytokines or chemokines. Based on this study, it was concluded that laser radiation of the MLS M1 system activates PBMCs to generate IL-1β and CCL3. We compared cytokine/chemokine concentrations in illuminated and non-illuminated cells and cells illuminated with different doses of radiation at various frequencies.

**Fig. 3:**
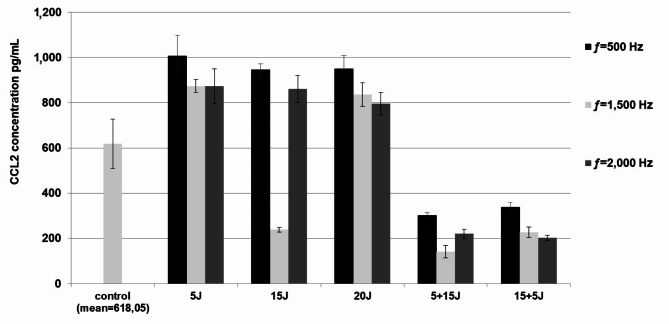
An ELISA test on supernatant samples evaluated the effect of PBMT on CCL2 synthesis in PBMCs. PBMCs after irradiation were cultured for 72 h at concentrations of 1 × 10^6^ cells/mL (control was not irradiated). Results are shown as the mean ± SD of three separate experiments. *p < 0.05.

There were no significant changes in CCL2 levels of PBMT-treated cells compared with the control. Still, a statistically significant difference in CCL2 concentration was observed after fractional application of laser radiation at a dose of 15 + 5 J with a frequency of 1,500 Hz (mean concentration ± SD: 141,75 ± 27,36 pg/mL) compared to cells irradiated with laser radiation at the following parameters: ƒ=500 Hz at energy doses of 5 J (1006,47 ± 92,73 pg/mL, *p* = 0,016), 15 J (944,85 ± 27,36 pg/mL, *p* = 0,045) and 20 J (950,15 ± 59,46 pg/mL, *p* = 0,04) (Fig. 3).

**Fig. 4:**
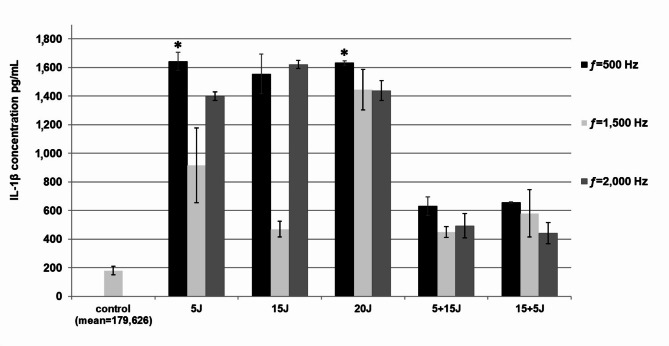
Effect of PBMT on IL-1β synthesis in PBMCs evaluated by ELISA test in supernatant samples. PBMCs after irradiation were cultured for 72 h at concentrations of 1 × 10^6^ cells/mL (control was not irradiated). Results are shown as the mean ± SD of three separate experiments. *p < 0.05.

The application of laser radiation from the MLS M1 system at a frequency of 500 Hz and energy dose of 5 J and 20 J resulted in a significant increase in IL-1β synthesis (respectively: 1643,14 ± 62,81, *p* = 0,029 and 1631,32 ± 16,71 pg/mL, *p* = 0,036) compared to the non-illuminated control (179,63 ± 29,63 pg/mL). It was also found that laser radiation at an energy dose of 5 J and 20 J dosed at 500 Hz resulted in the most significant increase in IL-1β synthesis compared to the other radiation parameters used. Still, this difference was not statistically significant (Fig. 4).

**Fig. 5:**
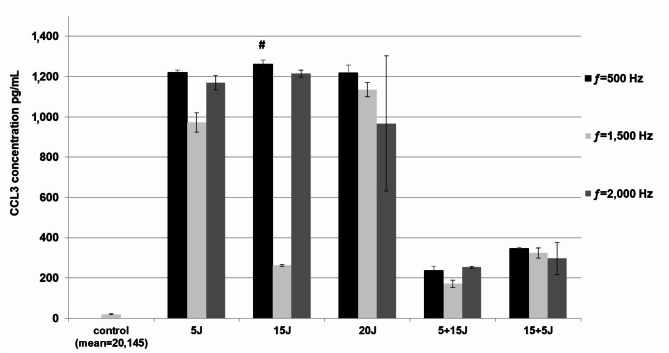
The effect of PBMT on CCL3 synthesis in PBMCs was evaluated using an ELISA test in supernatant samples. PBMCs after irradiation were cultured for 72 h at concentrations of 1 × 10^6^ cells/mL (control was not irradiated). Results are shown as the mean ± SD of three separate experiments. *p < 0.05, ^**#**^*p* < 0.01.

The study showed that laser radiation with an energy dose of 15 J and a frequency of 500 Hz (1261,52 ± 20,56 pg/mL, *p* = 0,01) caused a statistically significant increase in CCL3 synthesis compared to the control (20,15 ± 2,33 pg/mL. Furthermore, applying radiation with these parameters resulted in the most tremendous increase in CCL3 synthesis compared to all other radiation parameters tested. However, the difference in statistical significance occurred when compared to radiation with an energy dose of 5 + 15 J and a frequency of 1,500 Hz (170,82 ± 19,27 pg/mL, *p* = 0,029) (Fig. 5).

## Discussion

Our study evaluated the effect of PBMT on PBMC cytokine mRNA and protein expression that play a role in the pathogenesis of numerous diseases. PBMT is one of the therapies used to treat patients with this chronic autoimmune inflammatory disease, and there is substantial evidence that this treatment does not remain without influencing the immune cells. Presented results indicate that dual-wavelength MLS M1 system laser radiation applied in vivo dosed at 5 J and 20 J at a frequency of 500 Hz increased both mRNA expression and the synthesis level for IL-1β. Moreover, an increase in mRNA expression was observed after irradiation with an energy dose of 20 J (ƒ=1,500 and 2,000 Hz) and 5 + 15 J (ƒ=500 Hz), which were statistically significant. The results demonstrated that applying PBMT at energy doses of 5 + 15 J (ƒ=1,500 Hz) and 15 + 5 J (ƒ=2,000 Hz) resulted in a statistically significant increase in CCL2 and CCL3 gene expression. So PBMT can augment the pro-inflammatory activity of PBMCs. From a clinical point of view, this is not a desirable phenomenon. However, it should be considered that the effect obtained after applying the same radiation parameters in vitro and in vivo may differ due to additional absorbing or scattering elements and the depth of radiation penetration.

Some studies focusing on in vitro cell cultures have demonstrated that PBMT reduces the production of pro-inflammatory cytokines. Tamazoni et al. showed that laser irradiation (λ = 830 nm, continuous emission, power of 100 mW; energy density: 35.7 J/cm^2^, 107.1 J/cm^2^, and 321.4 J/cm^2^, irradiation time: 10, 30, and 90 s) significantly reduced levels of TNF-α, IL-1β and IL-6 at all assessed time points and energy densities. Of these, the 107.1 J/cm² energy density emerged as the most productive^38^. Additionally, Ebrahiminaseri et al. indicated that LLLT (450 nm, power of 75 mW, fluence 0.95 j/cm2) resulted in a reduction in pro-inflammatory cytokines (TNF-α, IL-6) in mouse embryonic fibroblast cells^39^.

In vivo studies have likewise demonstrated the anti-inflammatory properties of PBMT. Zanatta and colleagues also researched the effects of laser radiation on inflammatory processes, evaluating the concentrations of 38 pro- and anti-inflammatory cytokines. They showed that in patients with oral mucositis following the utilization of Low-Level Laser Therapy (LLLT) (λ = 970 nm, power 2.5 W, irradiance 200 mW/cm^2^, fluence 6 J/cm^2^, time 30 s) resulted in a reduction in the level of 16 out of 29 pro-inflammatory cytokines (including IL-1a, IL-3, IL-2Ra, IL-8, IL-16, CTACK, GRO-a, MIG, HGF, b-NGF, M-CSF, LIF and TNF-β). In contrast, four of nine anti-inflammatory cytokines were up-regulated (IL-4, IL-5, IL-10, IL-13)^8^. Wu et al. documented that in a mouse model of Alzheimer’s disease, the levels of IFN-γ and IL-10 in neural stem cells and T lymphocytes were upregulated following PBMT treatment (λ = 635 nm, energy density of 2 J/cm^2^, for 10 min daily for one month), which resulted in the alleviation of cognitive deficits^24^. De Brito et al. demonstrated a decrease of IL-4, IL-5, and IL-13 levels and an increase in the IL-10 levels in mice with chronic asthma following PBMT (λ = 660 nm, a power of 100 mW, energy dose of 5 J for 50 s/point)^28^. Neve’s team demonstrated the utilization of laser beams with a wavelength of 660 nm, power of 30 mW, and energy density of 50 J/cm^2^ significantly reduced pro-inflammatory levels of IL-6 in muscle tissue and the spinal cord. Also, the reduced level of IL-10 was restored after using PBMT at the site of carrageenan injection in the spinal cord^40^.

A potential explanation for this discrepancy compared to our findings may lie in the differences between in vivo and in vitro environments, such as systemic regulatory mechanisms, immune cell interactions, and tissue-specific responses in vivo conditions, which may not be fully replicated in vitro models. Additionally, the specific parameters of the PBMT applied, including wavelength, dosage, and irradiation mode (continuous or pulsed), could significantly influence the cellular response and contribute to the observed variations in pro-inflammatory cytokine production.

The results of our study did not corroborate previous findings regarding the effect of PBMT on the levels of pro-inflammatory cytokines, specifically IL-1β and CCL3, in PBMCs. This discrepancy may be attributed to the use of dual-wavelength laser emission. Analysis of the effects of PBMT on cytokine and chemokine concentrations indicated that energy doses of 5, 15, and 20 J had a notably more significant stimulatory impact on the levels of the investigated inflammatory mediators compared to fractionated doses (5 + 15 J and 15 + 5 J). However, not all results reached statistical significance. While specific fractionated doses led to increased mRNA expression of CCL2 and CCL3, these changes did not translate to the protein level. A review of the literature suggests that this phenomenon could be due to the ability of the MLS M1 laser system to enhance reactive oxygen species production while simultaneously inhibiting protein synthesis. In earlier own studies utilizing the MLS M1 system, authors observed that the production of ROS within cells increased over time, accompanied by a significant rise in intracellular calcium ion levels. Specifically, at 72 h following irradiation, intracellular Ca^2+^ concentrations were more than 50% higher than measurements taken immediately and 48 h after irradiation. Notably, cells exposed to pulsed mode irradiation exhibited a more pronounced increase in ROS and calcium levels over time than those exposed to continuous wave irradiation. Both the role of ROS and Ca^2+^ in the inflammatory process was confirmed^41^. Moreover, our mRNA expression and cytokine level measurements were conducted at least 72 h post-exposure of the cells to PBMT. Other studies have typically assessed these parameters at earlier time points, such as 12-, 24-, or 48-hours post-exposure. This temporal aspect of cellular responses to PBMT, particularly concerning the dynamics of ROS and Ca^2+,^ may help to explain the discrepancies observed between our results and those reported in other studies.

PBMCs produce a cytoprotective, regenerative, and immunomodulatory secretome upon exposure to oxidative stress-inducing factors. Current research suggests their potential application in regenerative medicine^42,43^. Scientists have shown that factors such as ionizing radiation, laser radiation, and physical exercise can result in functional alternations in PBMC, which are caused by an increased secretion of cytokines/chemokines or ROS. This can also lead to transient changes in mitochondrial energy metabolism^43–45^. It is also important to note the role played by PBMCs in the immune response not only in inflammatory or autoimmune diseases but also in response to the development of cancers. The cytokines these cells produce (specifically T lymphocytes) regulate the interaction between lymphocytes and tumor cells. They are involved in angiogenesis and apoptosis, including IL-2, IL-6, IL-8, IL-10, IL-12, IL-18, and TNF-α^46–48^. PBMCs have emerged as a source of biomarkers, including DNA methylation patterns, expression profile of mRNAs, microRNAs, and long non-coding RNA. These biomarkers have been associated with various diseases and conditions, including type 2 diabetes, chronic heart failure, coronary artery disease, Alzheimer’s disease, schizophrenia or RA^49–53^.

Although a trend was observed, the limited sample size necessitates further investigation to understand the influence of laser radiation parameters and optimize their selection to reduce the synthesis of pro-inflammatory mediators. Additionally, the role of power surface density in stimulating the pro-inflammatory activity of PBMCs, mainly through the mechanism of ROS production, should not be overlooked. It is plausible that simultaneous application of two laser wavelengths might require reducing power surface density to mitigate pro-inflammatory effects. However, this hypothesis warrants confirmation through additional research.

## Conclusion

Our findings suggest that dual-wavelength laser radiation from the MLS M1 system can modulate cytokine mRNA expression and stimulate PBMCs to produce pro-inflammatory cytokines and chemokines. Better understanding the specific effects of simultaneous dual-wavelength laser emission on inflammatory response mechanisms is crucial for optimizing radiation parameters to achieve the most effective therapeutic outcomes, both at the molecular and systemic levels.

## Materials and methods

### PBMCs isolation

Human PBMCs were isolated from buffy coats purchased from the Regional Centre for Blood Donation and Blood Treatment in Lodz (Poland) using the density gradient isolation technique as waste material. The Ethics Committee of the Medical University of Lodz, Poland, approved all procedures performed during the study (consent number: RNN/262/21/KE). 5 ml of PBS with 0.02 mM EDTA was added to a 5 ml buffer coat, stir gently, and applied to 5 ml of Histopaque-1077. Cells were centrifuged at 400 g. for 30 min at room temperature. The opaque cell layer was aspirated and transferred into a conical centrifuge tube, then washed in PBS (0.02mM) and EDTA trice. The number of cells was determined using a Bürker chamber.

### Cell culture

PBMCs were resuspended at 1 × 10^6^ cells/mL in medium with L-glutamine and sodium bicarbonate supplemented with 10% (v/v) fetal bovine serum (Gibco) and 1% antibiotics (100 U/mL penicillin, 100 mg/mL streptomycin; Sigma-Aldrich) in 12-well sterile (non-pyrogenic) polystyrene flat bottom plates (Corning). PBMCs were irradiated and cultured in a humidified incubator at 37 °C under a 5% CO_2_ atmosphere for 72 h. After incubation, cells were removed and centrifuged at 250 g for 7 min at 20 °C. The cell supernatants were subsequently frozen in aliquots at -80 °C.

### mRNA expression level by quantitative real-time amplification product analysis (qRT-PCR)

After 72 h of cell culture, PBMCs from 5 wells were pooled, and then RNA was isolated from 5 × 10^6^ PBMCs using TRI Reagent^®^ (Sigma-Aldrich). The concentration (A260) and purity (A260/A280) of RNA were measured using a NanoDrop 2000 spectrophotometer (Thermo Scientific, USA). Following the measurements, the RNA samples were promptly frozen at − 80 °C. Before analysis, RNA was reverse transcribed to cDNA using the High-Capacity cDNA Reverse Transcription Kit (Applied Biosystems, Foster City, California, USA), following the manufacturer’s protocol. The primer sequences were designed using Primer3 software and subsequently validated with Primer-BLAST. The primer sequences are presented in Table 1.

**Table 1:** The human primers sequence.

	Gene name	Primer sequence (5′-3′)
1	ACTB	Forward: CACCATTGGCAATGAGCGGTTC Reverse: CACCATTGGCAATGAGCGGTTC
2	CCL2	Forward: AGAATCACCAGCAGCAAGTGTCC Reverse: AGAATCACCAGCAGCAAGTGTCC
3	CCL3	Forward: ACTTTGAGACGAGCAGCCAGTG Reverse: ACTTTGAGACGAGCAGCCAGTG
4	IL1β	Forward: CCACAGACCTTCCAGGAGAATG Reverse: CCACAGACCTTCCAGGAGAATG
5	IL33	Forward: GCCTGTCAACAGCAGTCTACTG Reverse: GCCTGTCAACAGCAGTCTACTG
6	ST2	Forward: CTCTGTTTCCAGTAATCGGAGCC Reverse: CTCTGTTTCCAGTAATCGGAGCC

The expression of each gene was measured using SsoAdvanced Universal SYBR Green Supermix (Bio-Rad) in triplicate. The CFX Connect Real-Time PCR Detection System (Bio-Rad Laboratories) performed real-time quantitative amplification product analysis. Detailed conditions for the measurement are presented in the publication by Kozlowska et al.^54^. Changes in mRNA expression levels for individual proteins were calculated using Bio-Rad CFX MaestroTM Software using the ΔΔCt method and corrected by the transcript level of the housekeeping gene ACTB.

### Cytokine/chemokine synthesis measurement

Concentrations of selected pro-inflammatory cytokines/chemokines were measured using assays:

Human MCP-1 / CCL2 ELISA Kit (Biorbyt): range 15.6–1000 pg/mL, sensitivity less than 1 pg/mL,

Human MIP-1Alpha/CCL3 ELISA Kit (Biorbyt): range 15.6–1000 pg/mL, sensitivity less than 10 pg/mL.

Human IL-1β ELISA Kit (Biorbyt): range 15.625-1000pg/mL and sensitivity 6.6 pg/mL.

All procedures were conducted according to the manufacturer’s instructions. Results are shown as the mean ± SD of three separate experiments.

### Irradiation treatment

The laser irradiation was conducted at a controlled temperature of 22 ± 1 °C. The relative humidity in the irradiation environment was maintained at 50 ± 5%. The irradiation was performed in a dark room with minimal ambient light to prevent any interference with the laser treatment. To irradiation, MLS M1 emitting synchronized laser radiation at two wavelengths simultaneously (λ = 808 nm in continuous emission and λ = 905 nm in pulsed or continuous emission) has been used. Power density for continuous emission (CE) wavelengths 808 nm and pulsed emission (PE) 905 nm. Laser radiation energy was dosed in two ways: in one dose as a whole (5 J, 15 J, 20 J) and in a fractionated way (5 J + 15 J and 15 J + 5 J). The surface energy density (SED) was 1,59 − 6,37 J/cm2, and the surface area of the laser spot was 3,14 cm^2^. The distance between the laser probe and the bottom of the well was 10 mm. The radiation beam was positioned perpendicularly to the plate. Cells were irradiated immediately after placing in the 12-well sterile plates at 1 × 10^6^ cells/mL. PBMCs were not treated but incubated under the same conditions and were used as a control. The parameters of the PBMT are shown in Table 2.

**Table 2:** Parameters of the applied laser radiation.

Power density	The dosing method	Energy dose	Irradiation time
177 mW /cm^2^	(CE wave 808 nm, PE wave 905 nm, ƒ=500 Hz)	5 J	9 s
		15 J	27 s
		20 J	36 s
		5 J + 15 J	Light-on time 9s + 27s; off time 3s
		15 J + 5 J	Light-on time 27s + 9s; off time 3s
214 mW/cm^2^	(CE wave 808 nm, PE wave 905 nm ƒ=1,500 Hz)	5 J	8 s
		15 J	24 s
		20 J	32 s
		5 J + 15 J	Light-on time 8s + 24s; off time 3s
		15 J + 5 J	Light-on time 24s + 8s; off time 3s
230 mW/cm^2^	(CE wave 808 nm, PE wave 905 nm ƒ=2,000 Hz)	5 J	7 s
		15 J	21 s
		20 J	28 s
		5 J + 15 J	Light-on time 7s + 21s; off time 3s
		15 J + 5 J	Light-on time 21s + 7s; off time 3s

### Statistical analysis

The results were presented as mean ± standard deviation. The data obtained in the experiment were tested for normality with the Shapiro-Wilks test. Since the data sets were not normally distributed, the non-parametric ANOVA Kruskal—Walli’s test assessed the significance of differences. Post-hoc nonparametric pairwise comparisons were conducted using the Mann-Whitney U-test. A value α = 0.05 was taken as the level of statistical significance. The results were analysed using Statistica v. 13.3 software.

## Data Availability

The datasets used and/or analysed during the current study are available from the link https://doi.org/10.5281/zenodo.10624161.
